# Design and Implementation of a Contemporary Health Administration Program for Health Managers

**DOI:** 10.3389/fpubh.2021.735055

**Published:** 2021-10-04

**Authors:** Hanan Khalil, Chaojie Liu

**Affiliations:** Department of Public Health, School of Psychology and Public Health, La Trobe University, Melbourne, VIC, Australia

**Keywords:** pedagogy, health administration, health administration and leadership, health administration education, competence & performance

## Abstract

**Background:** While there are core competencies required in health administration programs, little is known about how they are taught in health administration programs to support/change practises. This discussion paper describes an educational innovation to design a contemporary Master of Health Administration program to meet the current needs of health managers in Australia based on evidence-based practise.

**Method:** A detailed gap analysis of health managers educational needs was undertaken with various stakeholders to design a contemporary health managers' program. Stakeholders surveyed in the course design included prospective students, international students' agencies, prospective employers, Alumni evaluation, mapping of health managers courses in Australia and faculty feedback. An integrative pedagogical approach was used to implement the program into action.

**Results:** Various themes were emerged from the stakeholder consultations including the importance of basic knowledge of key subjects and the significance of learning new skills such as strategic planning and emotional intelligence in the workplace. The integrative pedagogical approach used is based on adult teaching principles, which were identified by Knowles. The subjects in the new course incorporate several knowledge-based presentations along with interactive activities, including use of general ability-based outcomes to define learning opportunities, case-based and problem-based learning, experiential learning, and comprehensive assessments.

**Conclusion:** The results of this intensive consultation led to the design of a contemporary Master of Health Administration Course that included eight core subjects and multiple options of specialisations for students to choose from. Examples of specialisations include aged care and ageing, health promotion, data for decision making, public health, international development and Health Strategy and decision making.

## Background

Healthcare management programs play an essential role in preparing future managers in health services to deliver appropriate and efficient care in health services (1, 2). Although there are undergraduate degree courses in health administration programs, it has been widely recognised that postgraduate training is more adequate to prepare health management workforce. In general, postgraduate training courses offer students practical skills in addition to their existing foundation knowledge (3, 4). However, great variations exist in the contents and delivery of these courses. A study by Bonica et al. has used an open pedagogy to develop competency for health care programs (5). The authors argued that having open access resources available for students led them to enhance their soft skills and management. This methodology was complemented with assignments and discussion of outcomes. This teaching method relied on self-direction with the use and creation of open educational resources. The authors added that this method is well suited for health mangers as they are expected to be self-directing and have exploratory nature which appear to be effective for exercising the softer competencies, such as achievement orientation, information seeking, team leadership, and organisational awareness (5).

Despite the diverse background of health managers, empirical evidence shows that a set of essential management skills are required to enable achievement of high performance of a health organisation in relation to the organisation and its employees, consumers and other external stakeholders (6, 7). Health management and administration professional bodies in some countries have started to develop a unified management competency framework (4, 8). The competencies endorsed by these associations include components of leadership, knowledge of health and health care environment, business skills, communication and management skills and professional responsibilities (9, 10). These may also include social responsibilities from a population health perspective, addressing issues like cultural diversity and health disparity (11–13).

Over the years, health administration training programs have evolved to address several competencies highlighted by the various professional associations of health managers (4, 10). To date, however, only a limited published programmes have cited a basis of a pedogeological framework for their program. A study published by Abad Jorge et al. described pedagogical approaches for integrating cultural competency in a healthcare management program. The authors used this framework to address the health disparities amongst culturally diverse populations (8). The program included educational content on cultural competency, understanding and managing diversity, and the benefits of diversity leadership in healthcare. The authors used a variety of methods of teaching to cater for the various types of learners (8). More recently, a paper published by Caron et al. highlighted the use of population health pedagogy to address the evolving changes in health care challenges (14). The use of population health pedagogy includes the use of real data to assess the health requirements of a particular community; analyse and interpret diverse data sets; develop practical solutions to complex health issues based on evaluation and finally, successfully communicate to wide-ranging stakeholders.

A student-centred participatory approach is critical for students to obtain competencies required for health administration (8, 15). A recent integrative review reported various innovative pedagogical practises used in higher education aiming to increase students' engagement, motivation and critical thinking. Several approaches to increase reflection, higher level thinking, and deep learning are all essentials for students' learnings (16). Collaborative learning strategies where students learn from their peers and faculty are also encouraged as they resulted in higher engagement and conceptual understanding (17, 18). This is in addition to the use of various digital simulation tools ranging from videos to explain concepts, feedback provision and exist surveys to evaluate teaching (19, 20). The use of flipped classrooms was also encouraged in large classes due to its benefits in managing large numbers (21, 22). Having students access materials beforehand enabled them to come back with some ideas to discuss concepts with their peers and learn from each other through case studies and experiences from each other.

This paper describes the development of a contemporary Master of Health Administration (MHA) program in Australia involving an integrative pedagogical approach to implement the program into action. It is important to note that many of the health administration programs are still evolving and their curricula are constantly being updated to keep up with the evolving nature of health care and the challenging issues health managers face. Examples of these issues include the recent COVID 19 pandemic, rising costs of care and demands from consumers to deliver quality care in health services.

## Method

Re-design of an existing MHA course was conducted in 2020 through extensive stakeholder consultations. Both the existing and the revised courses went through accreditations from the Australasian College of Health Service Management in line with its management competency framework. The course is based on several competencies set by the Australasian College of Health Services Management which include the following: leadership, health and health care competencies, business skills, communications and relationship management and professional and social responsibility. This framework is based on competencies compiled by the Global Consortium (23).

### Gap Analysis and Contents Design

**Figure 1:**
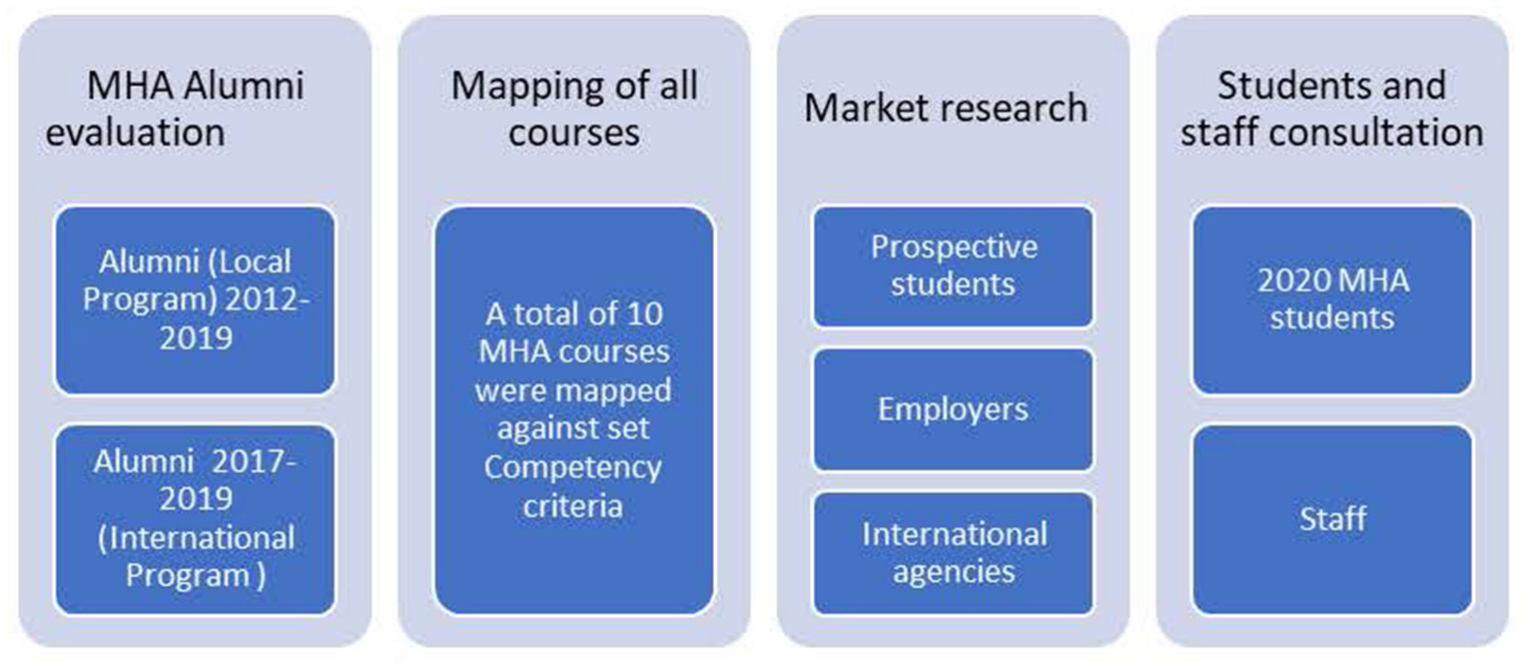
Details of the stakeholders involved in the Gap analysis.

A detailed gap analysis of health managers educational needs was undertaken with various stakeholders. The GAP analysis methodology is a relatively widely used approach for quality assurance processes in organisations that deliver services (24). However, it has been used only seldom in the University sector and the problems of its implementation include its difficult interpretation (25). Figure 1 shows the various stakeholders consulted in the gap analysis to generate the requirement for a contemporary health administration program. The Gap analysis consisted of a four-step approach; firstly, we identified the current state of the course by consulting with Master of Health Administration (MHA) Alumni who completed the course from 2012 to 2019. We have undertaken this step for both the local and the international programs run by the University. Ethics approval was obtained for the Alumni evaluation section of the project. Secondly, in order to identify what the new course design should entail, we have subcontracted market research undertaken by an independent consulting firm to engage potential employers, students and international agencies in a discussion about the future needs of health services managers in the workplace, including their opinions about the skills and knowledge required for them to engage in this evolving industry. Thirdly, we have identified the gaps in the current course through mapping the existing course against a set of competencies and similar courses offered in other Australian universities wherever their curriculum was made publicly available. The set of competencies were chosen based on the Royal Australian College of Medical Administrators (RACMA) framework, the Australasian College of Health Services Management (ACHSM) and the commission on Accreditation of Health Management Education (CAHM). These include: knowledge of health systems, systems improvement, finance, health policy, epidemiology, leadership, capstone/research project and work integrated learning. Student and external stakeholder expectations were summarised and discussed in our internal teaching and learning workshops. Finally, we have devised a plan for improvements of the current course in line with the adult teaching principles. This was undertaken with the consultation of current students and faculty staff.

### Implementation Plan of the Newly Designed Course

An integrative pedagogical approach was used to plan implementation of the program. This approach has been successfully used in the transition to online delivery of the MHA course. The approach is consistent with adult learning principles as mentioned by Knowles et al. emphasising use of general ability-based outcomes to define learning opportunities, case-based and problem-based learning, experiential learning and comprehensive assessments (26). Several strategies were employed to deliver this design, these included the extensive use of digital technologies for promoting engagement and motivation as seen in many courses, clear and succinct instructions to guide the learner to navigate the course content. This was also supported by aligning the outcomes of each subject with all the course outcomes as well as the topics delivered in each week and ensuring the assessments are relevant to the content delivered. Ensuring a culture of support and online presence by the faculty staff was also necessary to ensure guidance and appropriate and timely feedback. Finally, the use of real-life case studies and data to engage the learners and ensure the applicability of the content to the workplace was introduced in all assessments.

## Results

The results of the gap analysis are detailed below for each section of the gap analysis.

### MHA Alumni Evaluation

The findings of the MHA evaluation have been described in full elsewhere. Briefly, a total of 44 Alumni valued knowledge of project management, health services resources management, program evaluation and human resources as essential to their workplace. Skills addressing emotional strategic thinking and planning, resilience and emotional intelligence were also rated highly by all Alumni to be essential for the workplace. More than 90% of Alumni secured a promotion within their own organisation or were seconded to another organisation in a higher role. Furthermore, Alumni valued the flexible mode of delivery of the course to be essential as 80% were working in a full-time capacity and available to study part time due to their work and personal commitments. Recommendations to improve the course included having guest lectures from the industry and incorporating work placements as future suggestions to improve the course.

### Mapping of MHA Courses

A total of ten Health administration courses taught in Australian universities were identified and their core contents were mapped against the common areas identified by RACMA, ACHSM and CAHM. Each subject within each University curriculum was allocated one unit against the relevant theme. More than 75% Australian Health Administration courses had subjects in the themes identified by the accreditation bodies.

**Figure 2:**
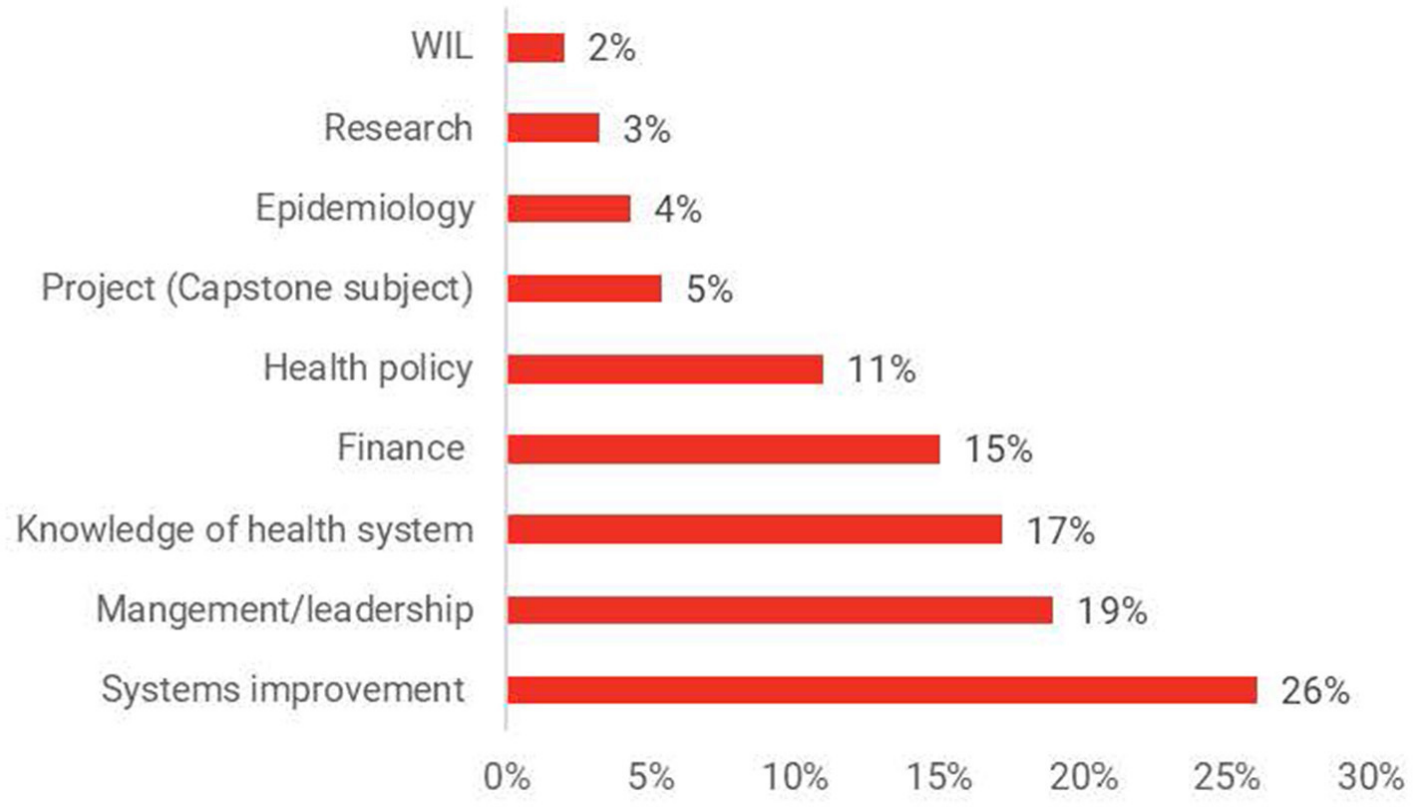
Proportion of themes in Australian Content of MHA courses.

Core subjects addressing systems improvement consisted of 26% of contents. Examples of these subjects include: program development and evaluation, evidence informed decision and using health care data for decision making. Management/leadership and knowledge of the health system each represented about 19% of the curriculum. Examples of subjects that align with these themes were health leadership and workforce management and the Australian health system respectively. Furthermore, 90% of all universities had a choice of either a capstone project or a research project. Only one University in Australia offered a placement for 12 weeks. Figure 2 shows the breakdown of topics within each theme by university.

### Market Research

One of the challenges in the re-design of the MHA course is to meet the diverse needs of students. In Australia a MHA course is usually taught over 1.5 (for students with a cognate degree in health) or 2 years (for students without a cognate degree in health). We proposed seven streams of specialisations (e.g., Applied Research, Health Promotion, Health Policy, Public Health, Community Engagement, Ageing in Society, and Ergonomics, Safety and Health) to meet the diverse need of the health sector. The main objectives of the marketing brief were to understand the value of specialisations in increasing employment prospects and/or career development, to understand overall appeal, industry alignment and impact on employment prospects of and to identify gaps in our specialisations' offer that would increase chances of employment of prospective students and to identify trends and industry skills future demand. The target groups for the market research included a total of ten participants consisting of employers in the health administration area, the university's Alumni, international agents recruiting students for the Master of Health Administration and prospective students. The results from the marketing confirmed the importance of specialisations that were seen as an important differentiator away from a “generic” masters' degree. The results also highlighted the importance of providing information regarding the reasons students choose one speciality over another and the differences between them. The findings from market research highlighted that there are three threads to potential specialisations including the following:

- Specialisations that push the MHA in particular into bigger picture spaces with a greater emphasis on strategic thinking within the health system.
- Specialisations that could form part of any of the Masters' degrees that reflect the likely long terms changes as a result of Covid-19.
- Specialisations that suit niche interests or very specific future careers.

### Current Students and Staff Consultation

A consultation of current students and staff took place separately before finalising the content of the new course to seek their opinions about the content and the mode of delivery of the course. Both groups endorsed the overall structure of the re-designed course but highlighted the importance of maintaining the flexible delivery of the course and the need to update the course content and the resources provided to the students. In line with the demands, the development of subjects embedded in this course incorporated previous feedback from students who undertook the subjects from previous the years. The subjects that were rated poorly by students were identified to be needed a significant amendment of course content, assessment, and delivery.

### Accreditation of the MHA Program

**Figure 3:**
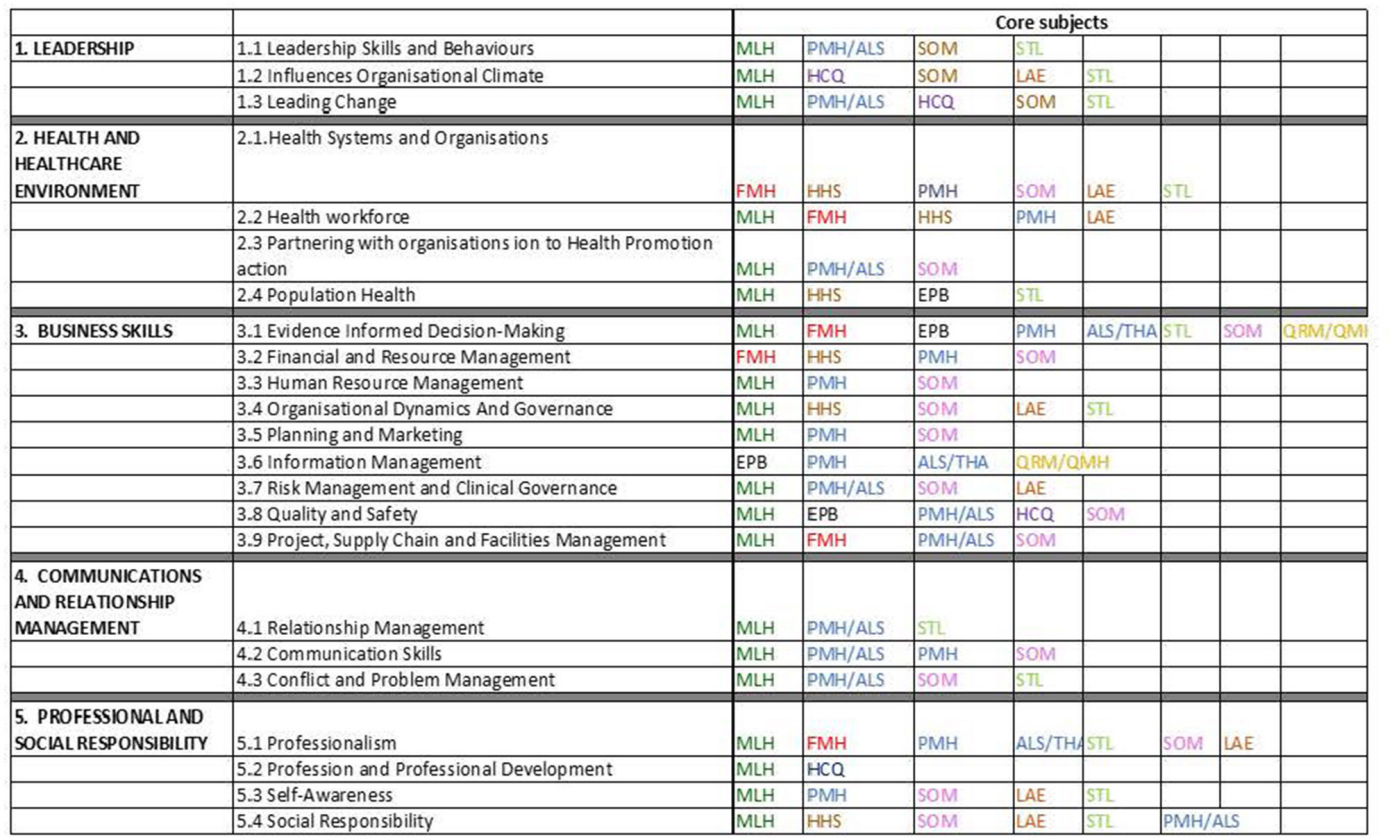
Mapping of subjects against core competencies of the accrediting organisation PHE5HHS -Health Systems; PHE5SOM – Strategy and operation management; PHE5FMH – Financial Management in Health Services; PHE5MLH- Management and leadership in Health; PHE5HCQ -Health Care Quality; PHE5EPB - Epidemiology and Biostatistics; PHE5LAE- Health law and Ethics; and PHE5STL- System thinking and Leadership; ALS-Action learning studies).

The re-designed MHA course was submitted for accreditation from the Australian College of Australasian College of health services management. This process required the course to demonstrate alignments of its subjects against the core competencies set by the college and assurance of effective arrangements of teaching delivery. This was the stage where further refinements of the subjects' content took place to ensure they are aligned with requirements of accreditation. The re-designed MHA course contains eight core subjects: PHE5HHS -Health Systems; PHE5SOM – Strategy and operation management; PHE5FMH – Financial Management in Health Services; PHE5MLH- Management and leadership in Health; PHE5HCQ -Health Care Quality; PHE5EPB - Epidemiology and Biostatistics; PHE5LAE- Health law and Ethics; and PHE5STL- System thinking and Leadership. These core subjects have covered all required core competencies including leadership, knowledge of health and healthcare environment, business skills, communications and relationships management and professional and social responsibilities as shown in Figure 3. The course also contains a choice of specialisation consisting of four subjects. Examples of specialisations include: digital health, data for decision making, ageing strategy, public health, health promotion, international development, advanced practise and applied research. The total course consists of 180 credit points where each subject is worth 15 credit points. One credit point is equivalent to about 10 h of study time for the students.

## Discussion

The design and implementation of the new MHA course was based best practise that included multiple stakeholders' consultation and evaluations from past students who are currently in practise. To our knowledge, this is the first course in Australia that was based on a gap analysis including engagement of multiple stakeholders, a robust pedagogy and the inclusion of several contemporary teaching pedagogy that relied on a solid blueprint of the course learning outcomes to align with the subjects intended learning outcomes and the assessments that included many real cases studies that promotes engagement from the learners and sharing case studies from their workplace.

The introduction of the specialisations has been a major change in the course and has been welcomed by employers and prospective students as it was highlighted to be needed by potential employers due the ongoing challenges that the health system is facing due to the pandemic, increasing costs of health care and shortages of trained workforce in the health sector that are suited for the upcoming challenges and the increased expected demands of health managers projects by the Australian Health Workforce agency (27, 28).

The specialisation arrangements can also be better tailored to the diverse work needs of students arising from different professional and cultural backgrounds. Examples of specialisations include digital health, data for decision making, ageing strategy, public health, health promotion, international development, advanced practise and applied research. Student profiles in the MHA course often mimic the diversity in workforce in the health industry. Management position descriptions can also vary significantly across different levels and different disciplines. One of the major changes in subject development in the re-designed course is to extend the focus on the Australian health system to international health systems. This will not only be attractive to the international students but also to the domestic students. It is important to recognise that countries can learn from each other.

The successful design and implementation of the course was undertaken using the triple C model, where a focus on consultation, collaboration and consolidation was employed (29, 30). The model was previously used to address complex organisations in health services (31, 32). However, it was found to be useful in implementing the course design and delivery. This step of the process relied on having a collaborative teaching team who had the knowledge and expertise in subject development and being innovative about how to align assessments and content with the learning outcomes of each subject and the course as a whole.

Health systems improvement is very challenging for many countries including the United States due to the increasing constraints on governments, increase focus on patient centred care, clinicians' overload and politicised workplaces. This study can be replicated in other countries to design bespoke health administration courses that are relevant and can address the needs of the changing health systems in various countries (33).

Future work will focus on evaluation of this course to meet the changing demands of the sector and the usefulness of the list of specialisations introduced into the new course. Identifying the popular specialisations offered will provide an insight into the industry demands and the support that future managers are needing to address their work challenges.

## Data Availability Statement

The original contributions presented in the study are included in the article/supplementary material, further inquiries can be directed to the corresponding author.

## Ethics Statement

The studies involving human participants were reviewed and approved by La Trobe University research Ethics Committee. The patients/participants provided their written informed consent to participate in this study.

## Author Contributions

HK designed and drafted the manuscript. CL has commented on it and provided input and feedback on the final copy. Both authors contributed to the article and approved the submitted version.

## Conflict of Interest

The authors declare that the research was conducted in the absence of any commercial or financial relationships that could be construed as a potential conflict of interest.

## Publisher's Note

All claims expressed in this article are solely those of the authors and do not necessarily represent those of their affiliated organizations, or those of the publisher, the editors and the reviewers. Any product that may be evaluated in this article, or claim that may be made by its manufacturer, is not guaranteed or endorsed by the publisher.
